# *Aedes albopictus* life table: environment, food, and age dependence survivorship and reproduction in a tropical area

**DOI:** 10.1186/s13071-021-05081-x

**Published:** 2021-11-07

**Authors:** Guzhen Cui, Saifeng Zhong, Tuquan Zheng, Zhangrui Li, Xu Zhang, Chuang Li, Elizabeth Hemming-Schroeder, Guofa Zhou, Yiji Li

**Affiliations:** 1 Key Laboratory of Tropical Translational Medicine of Ministry of Education, Hainan Medical University, Haikou, 571199 China; 2 Key Laboratory of Medical Microbiology and Parasitology of Education Department of Guizhou, School of Basic Medical Science, Guizhou Medical University, Guiyang, 550025 China; 3 Department of Pathogen Biology, Hainan Medical University, Haikou, 571199 China; 4 Program in Public Health, College of Health Sciences, University of California at Irvine, Irvine, CA 92617 USA; 5 Hainan Medical University-The University of Hong Kong Joint Laboratory of Tropical Infectious Diseases, Hainan Medical University, Haikou, 571199 China

**Keywords:** *Aedes albopictus*, Tropical area, Life table, Survivorship, Development time, Reproduction, Indoor, Shaded, Half-shaded

## Abstract

**Background:**

Environmental conditions affect the biology of mosquito vectors. *Aedes albopictus* is a major vector of many important diseases including dengue, Zika, and chikungunya in China. Understanding the development, fecundity, and survivorship of *Ae. albopictus* mosquitoes in different environmental conditions is beneficial for the implementation of effective vector control measures.

**Methods:**

*Aedes albopictus* larval and adult life-table experiments were conducted under natural conditions in indoor, half-shaded, and fully shaded settings, simulating the three major habitat types in Hainan Province, a tropical island in the South China Sea. Temperature, humidity, and light intensity were recorded daily. Larval rearing used habitat water and tap water, with and without additional artificial food. Development time, survivorship, pupation rate, and adult emergence rates were monitored. Adult mosquito survivorship and fecundity were monitored daily and reproductive rates were determined, and age-dependent survivorship and reproduction were analyzed.

**Results:**

The pupation time and male and female emergence times were significantly shorter in indoor conditions than in shaded and half-shaded conditions for both tap water with added food and habitat water with added food groups. For habitat water with added food, the shaded environment had the lowest pupation rate among the settings. For tap water with added food group, the shaded environment had the lowest pupation rate. The mean survival time of females was 27.3 ± 0.8 days in the indoor condition, which was significantly longer than that in the half-shaded (18.4 ± 0.6 days) and shaded (13.8 ± 1.2 days) conditions. Adult mortality was age-dependent, and the rate of change in mortality with age was not significantly different among different environmental conditions. The mean net replacement rate (*R*_0_) of female mosquitoes showed no significant difference among the three conditions, whereas the per capita intrinsic growth rate (*r*) in the shaded condition was 42.0% and 20.4% higher than that in the indoor and half-shaded conditions, respectively. Female daily egg mass was also age-dependent in all the settings, decaying exponentially with age.

**Conclusions:**

Our results imply that half-shaded conditions are likely the best natural condition for adult emergence and female reproduction, and food supply is crucial for larval development and pupation. The results provide new avenues for integrated mosquito management in indoor and outdoor areas, especially in half-shaded areas.

**Graphical Abstract:**

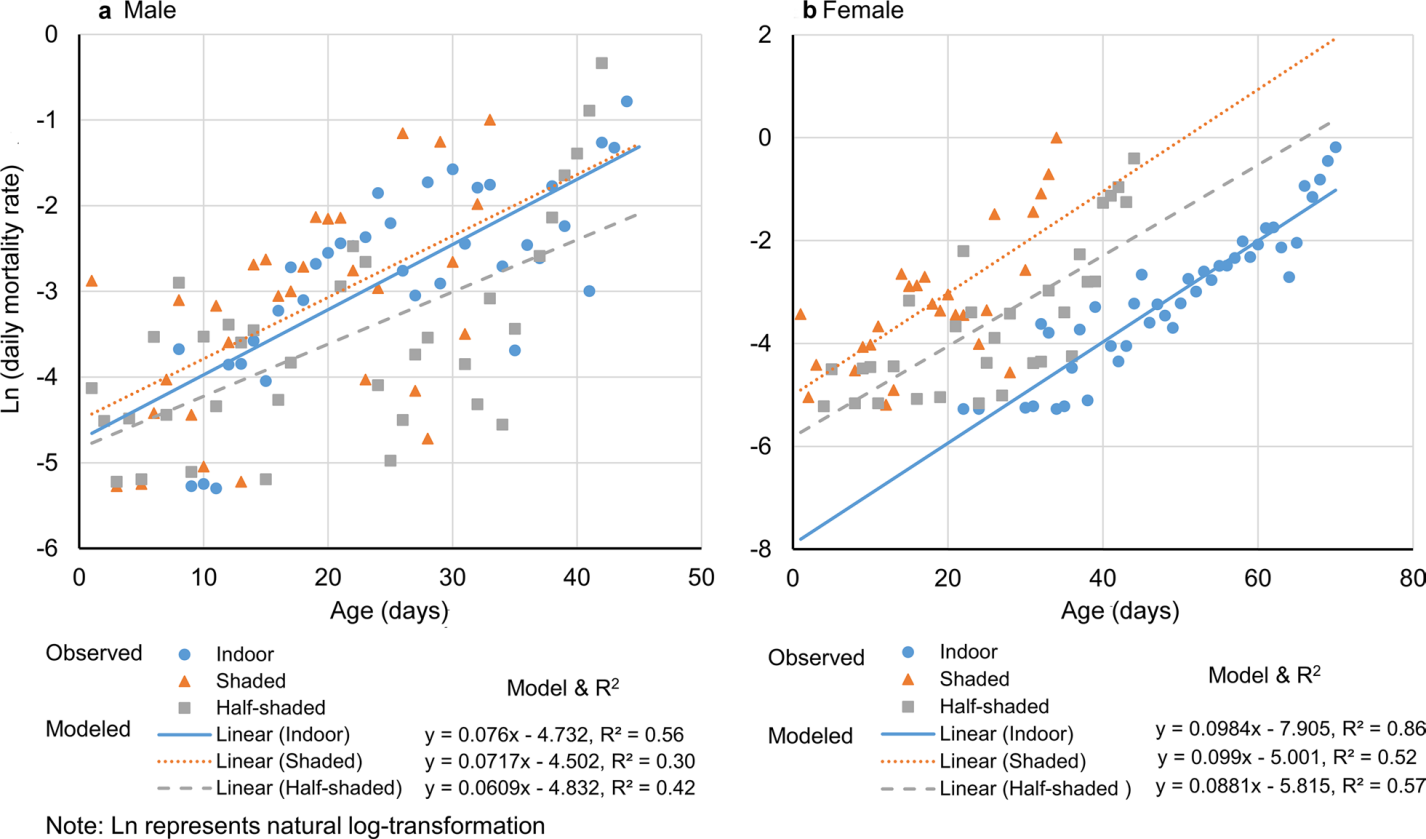

**Supplementary Information:**

The online version contains supplementary material available at 10.1186/s13071-021-05081-x.

## Background

*Aedes albopictus* is a strongly exophagic and exophilic mosquito with high mammalian affinity [1–3]. As one of the most invasive mosquito species, it has spread worldwide and emerged as a global public health threat [4]. *Aedes albopictus* is one of the major vectors of dengue, chikungunya, and Zika viruses, especially within the regions of Southeast Asia [5–7]. Control of *Aedes*-transmitted diseases depends largely on the control of the vectors [8]. Integrated and comprehensive descriptions of the mosquito life traits such as larval development time, adult survivorship, fecundity, and life expectancy in different environmental conditions are important for a better understanding of vector biology, which is necessary for effective vector control.

In the tropics and subtropics, potential *Ae. albopictus* larval habitats are diverse and include several types of containers, such as vases, flower pots, and retired tires in urban areas [9–13], as well as rubber plantations and agricultural fields (e.g. under banana plants) within the forested areas [14, 15]. Although habitat types may vary among different ecological settings, such as in different areas of urbanization [10, 16], these diverse aquatic habitats can generally be divided into the following four categories: (1) indoor environments, such as flower pots, jars, and water containers, which are common in the tropics and subtropics [17–20]; (2) semi-shaded environments, for example retired tires and rice fields [21, 22]; (3) nearly fully shaded areas, for example, aquatic habitats within banana fields or forested areas [14]; and (4) nearly fully open areas, such as drainage ditches and open containers [10, 23]. Similarly, adult *Ae. albopictus* can be found in diverse places, sometimes indoors although more often outdoors [24–26], including forested areas [27, 28]. Studies on *Anopheles* mosquitoes have found that the four aquatic environmental settings have different impacts on mosquito biology, such as larval development, adult survivorship, and reproductive potential [29–33].

These diverse environments may affect the microclimatic conditions as well as larval food supply, which in turn affects larval development, adult emergence, adult survivorship, and reproductive potential [14, 19, 31, 34]. For example, Alam and Tuno [35] found that extreme heat and/or extreme low humidity significantly reduced *Ae. albopictus* female reproductive capacity even if they could take blood meals. In addition to microclimatic conditions [30–32], studies of *Anopheles* mosquitoes found that food addition is important for immature mosquito development and pupation rates [29, 33, 36, 37]. For example, Munga et al. [33] found that adding food to the habitats in forested areas (nearly fully shaded) could increase *Anopheles gambiae* pupation rates from 2 to 23%. These previous studies suggest that both microclimatic conditions and food supply are important for larval development and mosquito reproduction. Although the impact of environmental conditions on mosquito life traits has been studied in life-table experiments, with many conducted under laboratory conditions, simulated natural conditions, semi-natural conditions, or even natural conditions [10, 30, 38], and some with in-depth details such as impact on gonotrophic time, reproductive potential, and vectorial capacity [31, 32, 39], it is not known how different environmental conditions affect *Ae. albopictus* life traits, such as larval development and adult survivorship.

The aim of this study was to evaluate the impact of different environmental conditions (indoor, half-shaded, and fully shaded) and food addition on larval development and adult survival of *Ae. albopictus* mosquitoes in a tropical area. The results not only provide new insights into the development and survival characteristics of this mosquito vector in tropical areas, but are also useful for implementing effective vector control strategies.

## Methods

### Study sites

The experiment was conducted in an urban area in Haikou City, Hainan Province, China. The area has a tropical climate, with an annual average temperature of 21.6 °C and annual precipitation of 1980 mm, which is ideal for the development and reproduction of most mosquito species [40]. The larval and adult *Ae. albopictus* life-table experiments were conducted in three settings: indoor, half-shaded, and fully shaded (Additional file 1: Figure S1). We selected an indoor environment because flower pots and water tanks are usually placed indoors or under the shade and are ideal for mosquito larval breeding in the study area. Larval rearing containers and adult rearing cages were set inside the large microcosms. Experimental microcosms were laid on the ground under the three environmental settings (Additional file 1: Figure S1). All microcosms were covered with insect-proof mesh to prevent mosquito escape or entry. All experiments, including indoor experiments, were conducted in uncontrolled conditions, i.e., temperature, humidity, and light were all under natural conditions. Experiments were not conducted in a fully open setting due to the excessive heat in the area during the middle of summer, which does not allow for *Ae. albopictus* survival in fully open settings.

### Source of mosquitoes

In the first 2 weeks of July 2019, field strains of *Ae. albopictus* larvae (regardless of stage) were collected from > 20 breeding habitats in several residential areas in Longhua District (19°53′51″ N, 110° 20′12″ E) of Haikou City, Hainan Province, China. Field-collected larvae were placed into a bucket and transferred to the study sites. Larvae were reared in microcosms in the three settings as described above, where abiotic conditions were all natural. Emerged adults were mixed and allowed to mate freely within the experimental cages. This mixing of adults from different habitats reduced the bias due to differences in the larval source and inbreeding. *Aedes albopictus* adults were identified morphologically under a stereomicroscope using taxonomic keys [41]. The mosquitoes were reared to F1 eggs under semi-field conditions. Eggs were mixed in the rearing basins, then reared in natural habitat water until the start of experiments.

### Larval life-table experiments

For each experiment, 30 newly hatched (< 24 h old) larvae were placed in each plastic bucket (16 cm caliber, 16 cm bottom diameter, and 18 cm height). The bucket was set insides the microcosms. Larvae were reared under three rearing conditions: (1) 1.5 L tap water (dechlorinated) stored overnight with added food; (2) 1.5 L habitat water with added food; and (3) 1.5 L habitat water without added food. Five replicates were conducted for each of the three rearing conditions in each of the indoor, half-shaded, and shaded settings. Each day, the surviving larvae were counted, and their stage was recorded. A total of 1350 larvae were used: (30 larvae) × (3 rearing conditions) × (3 environmental settings) × (5 replicates). In the food addition conditions, larvae were fed 1:1 yeast/fish food (by weight) every day, at an average of 0.1 g per bucket per day for all experiments. Water levels in the bucket were checked daily and maintained by adding the same type of water as needed. Water temperature and light intensity were measured using HOBO MX2202 data loggers (Onset Computer Corp., Bourne, MA, USA) placed about 2 cm below the water surface. The pupae were counted and removed daily. The emergence time of each mosquito (male and female) was recorded daily.

### Adult life-table experiments

Newly emerged (< 24 h) adults were used in the adult life-table experiments, with protocols similar to those described in previous studies [30, 34]. Briefly, 40 female and 40 male adult mosquitoes within 24 h post-emergence were placed in a microcosm (32.5 cm × 26.5 cm × 26.5 cm). The microcosm was covered with nylon mesh to prevent the mosquitoes from escaping. Five replicates were used for each of the three environmental settings. The mosquitoes were provided with 10% glucose daily, and from day 5 and every 7 days thereafter, a mouse was placed in each cage for approximately 3 h for blood-feeding of mosquitoes. The cages were examined daily to count the number of living and dead mosquitoes, and the dead mosquitoes were removed. Eggs laid in each cage were collected using moist filter paper and counted daily under a microscope. HOBO MX2301A and MX2202 data loggers (Onset Computer Corp., Bourne, MA, USA) were placed inside the cages to record hourly temperature, relative humidity, and light intensity during the entire duration of the experiment.

### Egg hatching rate observation

Briefly, 100 eggs collected from the field within 7 days were placed in a 200-ml plastic cup with the addition of 100 ml tap water (dechlorinated) and stored overnight. The cup was covered with nylon mesh to prevent the emerged mosquitoes from escaping. Five replicates (500 eggs in total) were used for each of the three environmental settings. The cups were examined daily until day 7 to count the number of hatched larvae, and larvae were removed daily.

### Data analysis

One-way analysis of variance (ANOVA) was used to determine the differences in larval development time, emergence rate, adult mosquito survival time, and average number of eggs laid per female during its life-time in the indoor, half-shaded, and shaded settings and between different food supply status under different settings. The post hoc Tukey honestly significant difference (HSD) test was used to determine the significance of differences among the three settings. Daily average temperature, light intensity, and relative humidity were calculated from the hourly records.

Adult survivorship was evaluated using Kaplan–Meier survival analysis [36]. The log-rank test was used to compare the difference in survival curves between different settings. One-way ANOVA with post hoc Tukey HSD test was used to determine the differences in water temperature, water light intensity, air temperature, air light intensity, relative humidity, larval development time, emergence rate, adult mosquito survival time, egg hatching time, and daily mean production (log-transformed eggs/female/day) among the indoor, shaded, and half-shaded settings. Student’s *t*-test was used to compare differences in the rates of change in age-dependent mortality and age-dependent egg mass between different experimental settings. All statistical analysis was carried out using JMP 9.0 statistical software (JMP, SAS Institute Inc., Cary, NC, USA).

## Results

### Variations in the experimental conditions

#### Water temperature and light intensity during the larval experiments

Water temperature and light intensity under the shaded, half-shaded, and indoor settings are shown in Table 1. The temperature in indoor (29 °C) settings was significantly higher than that in the shaded (27.2 ºC) and half-shaded (27.6 °C) settings (ANOVA, *F*_(2, 147)_ = 66.68, *P* < 0.0001; Tukey HSD, *P* < 0.05). However, in our indoor environment, we did not use any artificial light source, and thus relied solely on natural light, which resulted in the lowest light intensity in the indoor setting in all experiments (Table 1). Among the three treatment groups, the light intensity in the half-shaded setting was significantly higher than that in the other two settings (ANOVA, *F*_(2, 1101)_ = 262.74, *P* < 0.0001; Tukey HSD, *P* < 0.05).

**Table 1:** Mean water temperature and light intensity during the larval experiments

Development condition	Study site	Temperature (°C) (mean ± SE)^†^	Light intensity (lux) (mean ± SE)^†^
Tap water + food			
	Shaded	27.2 ± 0.1 a	56.3 ± 5.7 a
	Half-shaded	27.4 ± 0.1 a	117.8 ± 19.0 b
	Indoor	29.3 ± 0.1 b	13.6 ± 2.0 c
Habitat water			
	Shaded	28.0 ± 0.1 a	80.2 ± 8.7 a
	Half-shaded	27.9 ± 0.1 a	154.9 ± 13.0 b
	Indoor	29.6 ± 0.1 b	12.8 ± 0.6 c
Habitat water + food			
	Shaded	20.8 ± 0.6 a	35.8 ± 5.1 a
	Half-shaded	20.6 ± 0.6 a	320.0 ± 37.7 b
	Indoor	22.8 ± 0.5 b	7.8 ± 2.0 a

^†^Tukey HSD comparison of temperature and light intensity among the three settings for the same treatment. Numbers connected with different (same) letters indicate significant (non-significant) differences among the three settings

#### Air temperature, relative humidity, and light intensity in the adult experiments

**Fig. 1:**
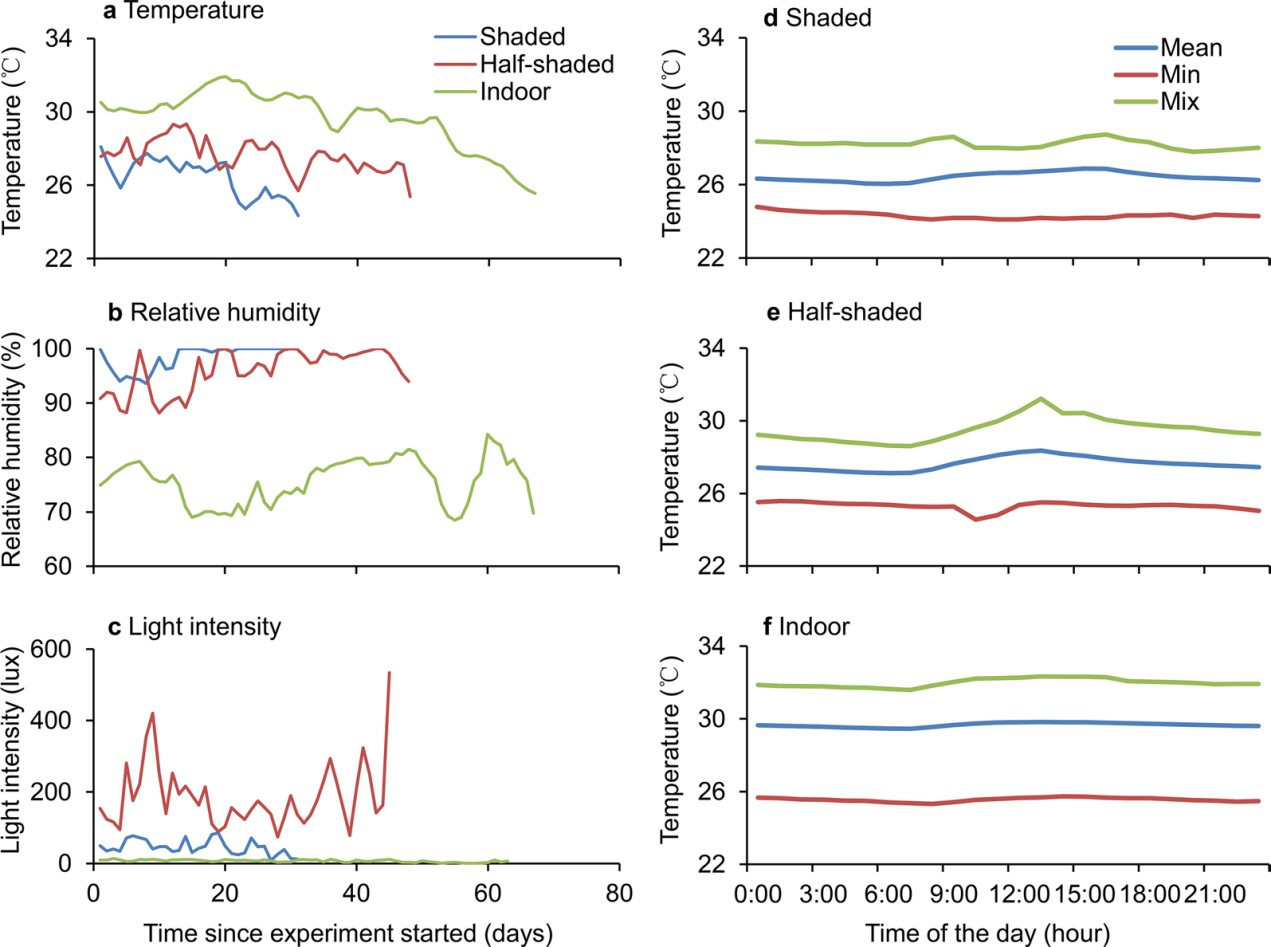
Air temperature, humidity, and light intensity in adult *Aedes albopictus* experiments in the three study settings. **a** Daily average temperature. **b** Daily average relative humidity. **c** Daily average light intensity. **d**–**f** Hourly average temperature in shaded, half-shaded, and indoor settings, respectively

Air temperature, relative humidity, and light intensity for the adult experiments are shown in Fig. 1 and Table 3. The temperature indoors was significantly higher than that in the shaded and half-shaded settings (ANOVA, *F*_(2, 143)_ = 79.48, *P* < 0.0001; Tukey HSD, *P* < 0.05). The relative humidity in shaded conditions was significantly higher than that in the other settings (ANOVA, *F*_(2, 143)_ = 596.31, *P* < 0.0001; Tukey HSD, *P* < 0.05). The light intensity in the half-shaded area was significantly higher than that in the other settings (ANOVA, *F*_(2, 134)_ = 158.50, *P* < 0.0001; Tukey HSD, *P* < 0.05).

### Egg hatching rate and time

**Fig. 2:**
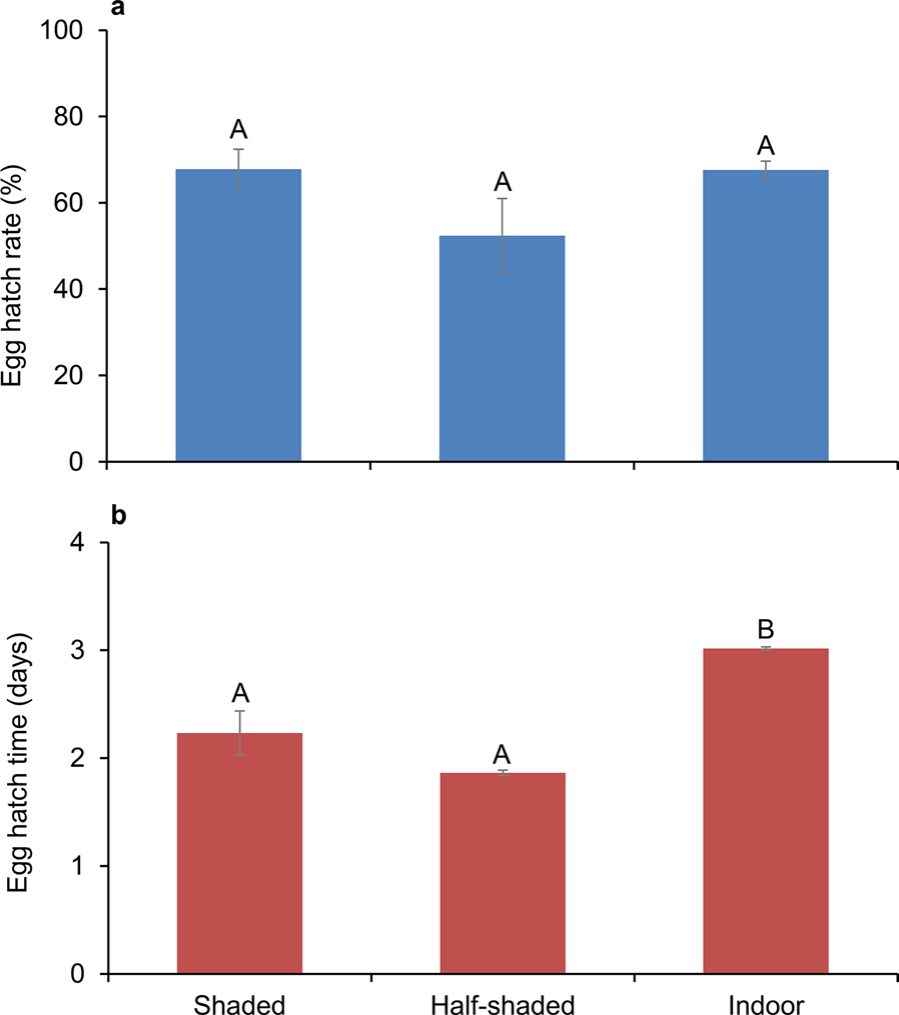
Egg hatching rate and hatching time of *Aedes albopictus* in the three study settings. **a** Egg hatching rate of *Ae. albopictus*. **b** Egg hatching time of *Ae. albopictus*. Error bars represent standard error of the mean. Bars labeled with different letters within the same panel are significantly different from each other (*P* < 0.05)

The egg hatching rates in the shaded, half-shaded, and indoor settings were 67.8% ± 4.6%, 52.4% ± 8.6%, and 67.6% ± 2.1%, respectively (ANOVA, *F*_(2, 12)_ = 2.36, *P* = 0.137) (Fig. 2a). However, the egg hatching time under indoor conditions was statistically longer than that under both the shaded and half-shaded conditions (ANOVA, *F*_(2, 12)_ = 24.85, *P* < 0.0001; Tukey HSD, *P* < 0.05) (Fig. 2b).

### Immature and adult development time

In the tap water with added food group, the average larval-to-pupal development times were 6.0 ± 0.1 days, 6.0 ± 0.1 days, and 5.2 ± 0.1 days for the shaded, half-shaded, and indoor settings, respectively (Table 2). The larval-to-pupal development time under indoor conditions was significantly shorter than that under shaded and half-shaded conditions (ANOVA, *F*_(2, 426)_ = 54.32, *P* < 0.0001; Tukey HSD, *P* < 0.05). In most cases, female and male adult development time in indoor settings was significantly shorter than that under shaded and half-shaded settings (female development time: ANOVA, *F*_(2, 129)_ = 9.65, *P* = 0.0001; Tukey HSD, *P* < 0.05; male development time: ANOVA, *F*_(2, 136)_ = 42.27, *P* < 0.0001; Tukey HSD, *P* < 0.05) (Table 2). In the habitat water without added food group, larval-to-pupal development time and female and male adult development time were long compare to the other two treatments, and they were not significantly different among shaded, half-shaded, and indoor settings (larval-to-pupal development time: ANOVA, *F*_(2, 62)_ = 0.54, *P* = 0.583; female development time: ANOVA, *F*_(2, 19)_ = 0.23, *P* = 0.8003) (Table 2). For both the habitat water with added food and tap water with added food groups, the average larval-to-pupal development time and female and male adult development time in the indoor setting were significantly shorter than that in the shaded and half-shaded settings (habitat water with added food group: average larval-to-pupal development time: ANOVA, *F*_(2, 398)_ = 40.68, *P* < 0.0001, Tukey HSD, *P* < 0.05; female development time: ANOVA, *F*_(2, 192)_ = 42.68, *P* < 0.0001, Tukey HSD, *P* < 0.05; male development time: ANOVA, *F*_(2, 201)_ = 54.69, *P* < 0.0001, Tukey HSD, *P* < 0.05 / tap water with added food group: average larval-to-pupal development time: ANOVA, *F*_(2, 426)_ = 54.32, *P* < 0.0001, Tukey HSD, *P* < 0.05; female development time: ANOVA, *F*_(2, 129)_ = 9.65, *P* = 0.0001, Tukey HSD, *P* < 0.05; male development time: ANOVA, *F*_(2, 136)_ = 42.27, *P* < 0.0001, Tukey HSD, *P* < 0.05).

**Table 2:** *Aedes albopictus* development time under different treatments in different settings

Treatment	Location	Immature stage		Female		Male	
		*N* pupae	Mean ± SE^†^	*N*	Mean ± SE^†^	*N*	Mean ± SE^†^
Tap water + food							
	Shaded	141	6.0 ± 0.1 a	33	8.5 ± 0.1 a	27	8.6 ± 0.2 a
	Half-shaded	141	6.0 ± 0.1 a	36	8.0 ± 0.2 ab	43	8.3 ± 0.2 a
	Indoor	147	5.2 ± 0.1 b	63	7.6 ± 0.1 b	69	6.9 ± 0.1 b
Habitat water							
	Shaded	13	28.6 ± 2.4 a	2	29.5 ± 7.5a	2	29.0 ± 8.0
	Half-shaded	42	25.4 ± 1.8 a	18	27.8 ± 2.6a	13	29.5 ± 2.4
	Indoor	10	24.3 ± 3.5 a	2	33.0 ± 2.0a	1	36.0
Habitat water + food							
	Shaded	125	7.5 ± 0.1 a	61	11.6 ± 0.2 a	62	11.6 ± 0.2 a
	Half-shaded	144	7.2 ± 0.1 a	68	12.2 ± 0.2 a	74	11.2 ± 0.2 a
	Indoor	132	6.3 ± 0.1 b	66	10.0 ± 0.2 b	68	9.2 ± 0.2 b

*N*: number of emerged females or males. Development times of females and males for the habitat water without added food treatment were not compared due to the small number of individuals

^†^Tukey HSD comparison of development time among the three settings for the same treatment. Numbers connected with different (same) letters indicate significant (non-significant) differences among the three settings. Comparison was not done for male development time for habitat water experiments because only one male emerged in indoor experiments

### Pupation and adult emergence rates

**Fig. 3:**
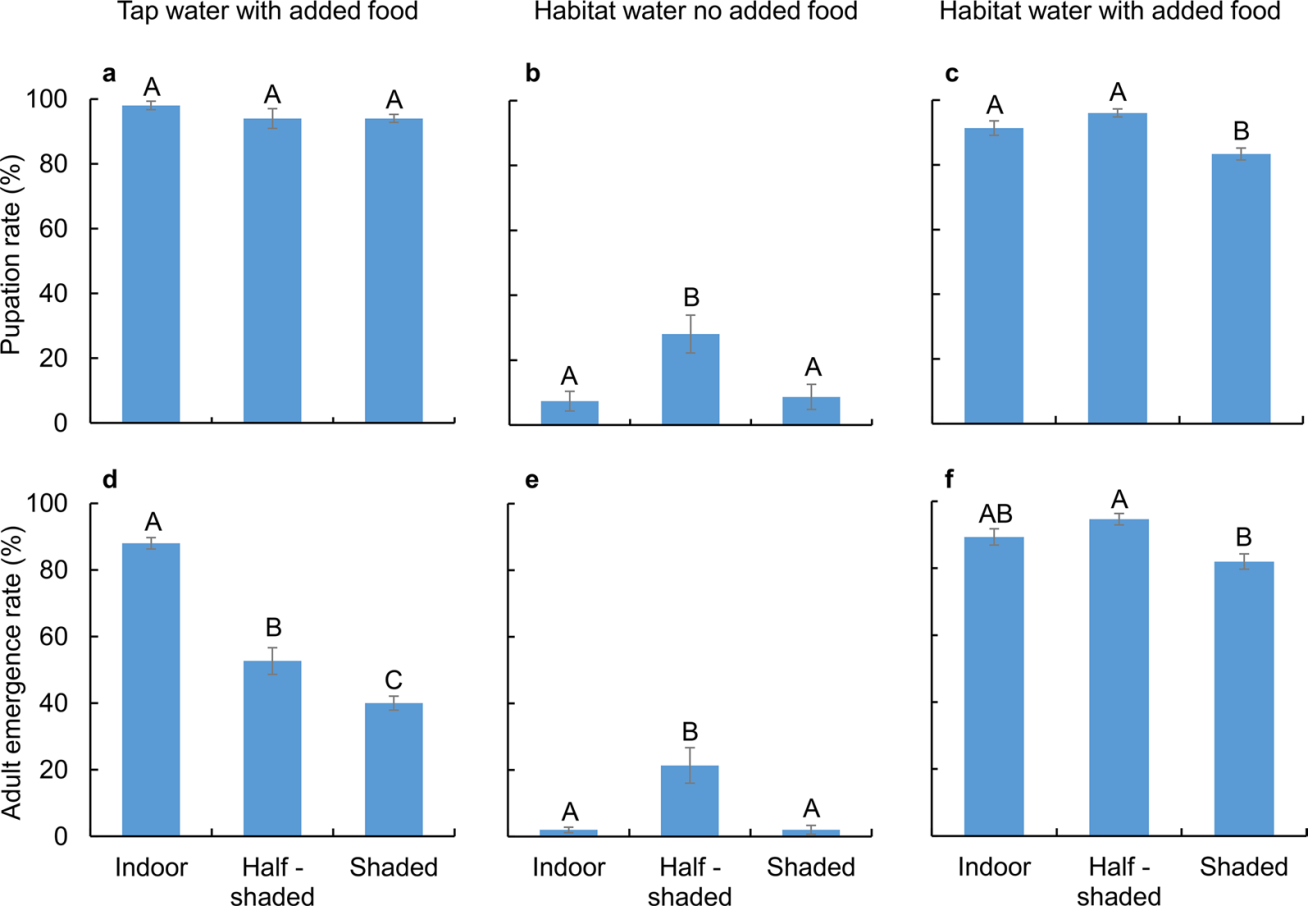
*Aedes albopictus* pupation (top panel) and emergence (bottom panel) rates under different treatments in the three study settings. **a**, **d** Tap water with added food treatment. **b**, **e** Habitat water with no added food treatment. **c**, **f** Habitat water with added food treatment. Error bars represent standard error of the mean. Bars labeled with different letters within the same panel are significantly different from each other (*P* < 0.05)

In the tap water with added food group, no significant differences in the pupation rates were observed among the three study settings (ANOVA, *F*_(2, 12)_ = 1.18, *P* = 0.345) (Fig. 3a). In contrast, within the same treatment group, the adult emergence rates differed significantly among different settings, with the indoor setting having the highest emergence rates (88.0 ± 1.7%) and the fully shaded setting having the lowest emergence rates (40.0 ± 2.1%) (ANOVA, *F*_(2, 12)_ = 79.56, *P* < 0.0001; Tukey HSD, *P* < 0.05) (Fig. 3d). In the habitat water without added food group, both pupation rates and adult emergence rates were < 30%, and the half-shaded setting had the highest pupation (28.0 ± 5.8%) and adult emergence (21.3 ± 5.3%) rates (pupation rate: ANOVA, *F*_(2, 12)_ = 6.87, *P* = 0.0103; Tukey HSD, *P* < 0.05; adult emergence rate: ANOVA, *F*_(2, 12)_ = 12.10, *P* = 0.0013; Tukey HSD, *P* < 0.05) (Fig. 3b and e). Once food was added to the habitat water, both pupation and adult emergence rates increased markedly, and their differences among settings became less significant (ANOVA, Tukey HSD, at significance level of 0.05) (Fig. 3c and f).

### Effects of different environmental conditions on adult mosquito survivorship

**Fig. 4:**
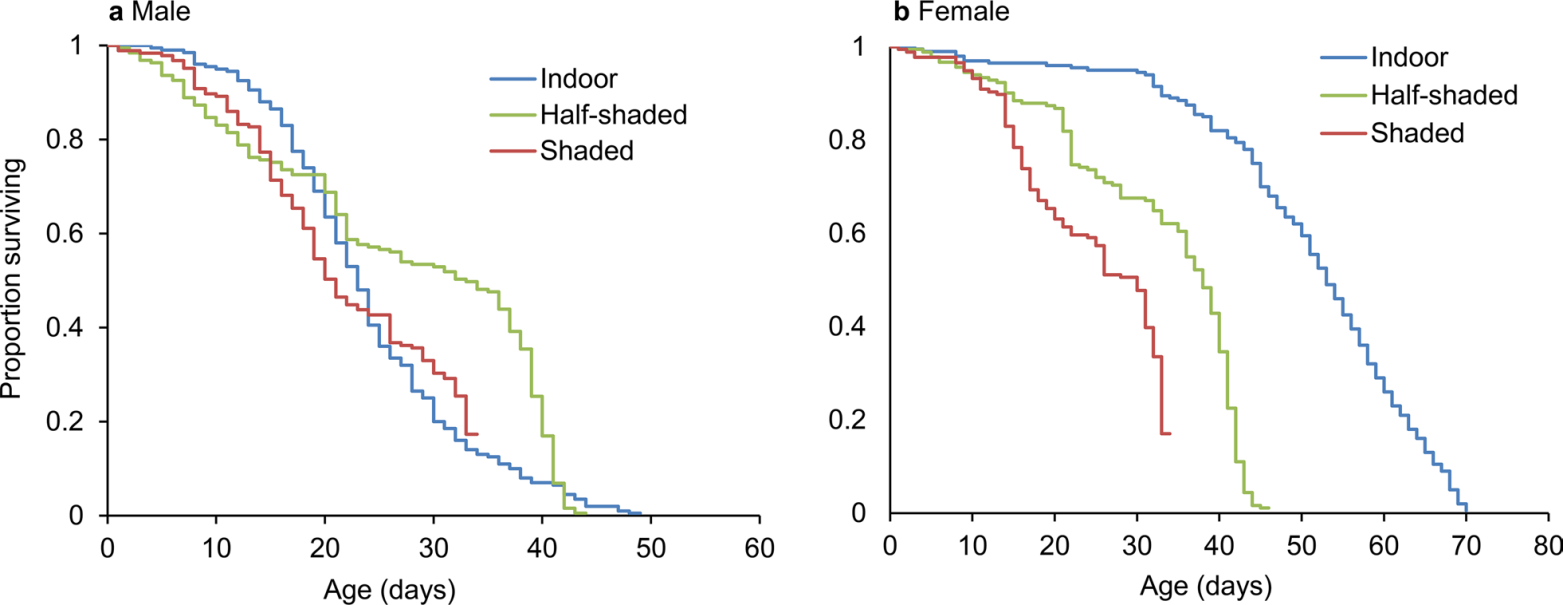
Kaplan–Meier plot of survivorship dynamics of *Aedes albopictus* in the three study settings. **a** Male mosquito. **b** Female mosquito

Both males and females had significantly different survival patterns under different environmental settings (log-rank tests, all *P* < 0.05) (Fig. 4). The mean longevity of male mosquitoes was 17.3 ± 0.6 days in the half-shaded setting, which was significantly longer than that in the indoor (13.8 ± 0.3 days) and shaded (12.7 ± 1.2 days) settings (ANOVA, *F*_(2, 12)_ = 8.36, *P* = 0.0053; Tukey HSD, *P* < 0.05; Table 3). The mean longevity of females was 27.3 ± 0.8 days under the indoor condition, which was significantly longer than that under the half-shaded (18.4 ± 0.6 days) and shaded (13.8 ± 1.2 days) conditions (ANOVA, *F*_(2, 12)_ = 60.06, *P* < 0.0001; Tukey HSD, *P* < 0.05; Table 3).

**Table 3:** Survival and reproductive characteristics of *Aedes albopictus* in different settings

Parameters	Detail	Indoor	Shaded	Half-shaded
Mean air temperature (℃)		29.7 ± 0.2 a	26.4 ± 0.2 b	27.6 ± 0.1 c
Mean air humidity (%)		75.6 ± 0.5 a	98.4 ± 0.4 b	95.9 ± 0.6 c
Mean air light intensity (lux)		7.0 ± 0.5 a	45.5 ± 3.8 b	188.1 ± 13.5 c
Mean survival time (days)	Male	13.8 ± 0.3 a	12.7 ± 1.2 a	17.3 ± 0.6 b
	Female	27.3 ± 0.8 a	13.8 ± 1.2 b	18.4 ± 0.6 c
Daily survival rate	Male	0.92 ± 0.01 a	0.92 ± 0.01 a	0.94 ± 0.01 a
	Female	0.95 ± 0.01 a	0.93 ± 0.01 a	0.95 ± 0.01 a
Male survival model parameters	Intercept	0.009	0.011	0.008
	Slope	0.076 a	0.072 a	0.061 a
Female survival model parameters	Intercept	0.007	0.003	0.0004
	slope	0.098 a	0.099 a	0.088 a
Longest survival time of females (days)		70	34	46
Female productive duration (days)		51.6 ± 0.75 a	29.2 ± 1.11 b	39.2 ± 0.37 c
Female daily mean production (eggs)		5.3 ± 1.2 a	12.4 ± 1.8 b	18.2 ± 1.7 b
*R*_0_^†^		61.7 ± 3.0 a	54.5 ± 8.0 a	74.2 ± 5.9 a
*r*^§^		0.19 ± 0.01 a	0.32 ± 0.02 b	0.25 ± 0.02 c

Numbers indicate mean ± SE. Tukey HSD comparison of parameter values among the three settings. Numbers connected with different (same) letters indicate significant (non-significant) differences among the three settings

^†^*R*_0_ is the mean net replacement rate (number of offspring per female per generation)

^§^*r* is intrinsic per capita growth rate (number of offspring per female per day)

**Fig. 5:**
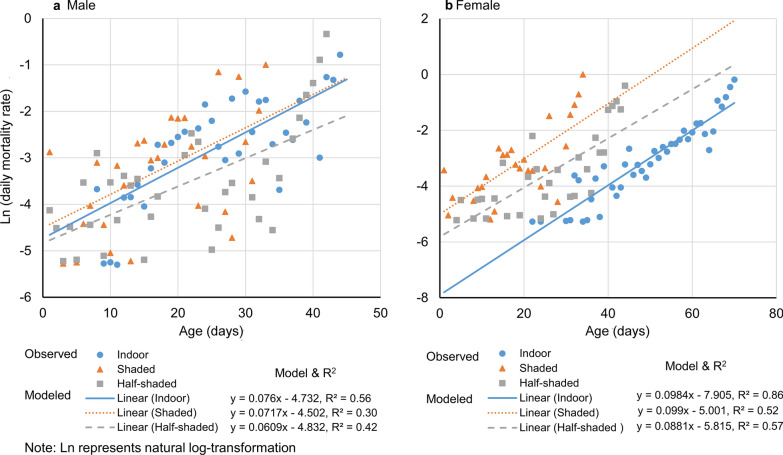
Age-specific *Aedes albopictus* mortality in three study sites. **a** Male mosquito. **b** Female mosquito. The value of *r*^2^ represents the proportion of variance in age-specific mortality explained by age

We found that both male and female adult mosquito mortality is age-dependent regardless of environmental conditions (Fig. 5). Significant positive values of slope (the rate of change in mortality with age) in the model suggested that the mortality rate was higher in older mosquitoes than in the younger ones (Fig. 5, Table 3). For female mosquitoes, the rates of change in mortality with age were similar between the shaded and half-shaded conditions (*t*-test, *t*_(53)_ = 0.44, *P* = 0.660), marginally different between indoor and half-shaded conditions (*t*-test, *t*_(73)_ = 1.97, *P* = 0.053), and significantly higher indoors than in shaded areas (*t*-test, *t*_(66)_ = 2.02, *P* = 0.048) (Table 3). However, for male mosquitoes, the rates of change in mortality with age were similar among the three settings (*t*-tests, all *P* > 0.05) (Table 3).

### Effects of different environmental conditions on mosquito reproduction

Female reproductive duration was the longest in the indoor setting (51.6 ± 0.75 days), followed by the half-shaded setting (39.2 ± 0.37 days), and shortest under the shaded conditions (29.2 ± 1.11 days) (ANOVA, *F*_(2, 12)_ = 194.72, *P* < 0.001; Tukey HSD, *P* < 0.05, Table 3). In contrast, female daily mean production, measured as average eggs laid per female per day, was the lowest under the indoor conditions, similar under outdoor conditions, but marginally non-significant in daily mean production among different settings (ANOVA, *F*_(2, 96)_ = 3.03, *P* = 0.053; Tukey HSD, *P* > 0.05, Table 3).

**Fig. 6:**
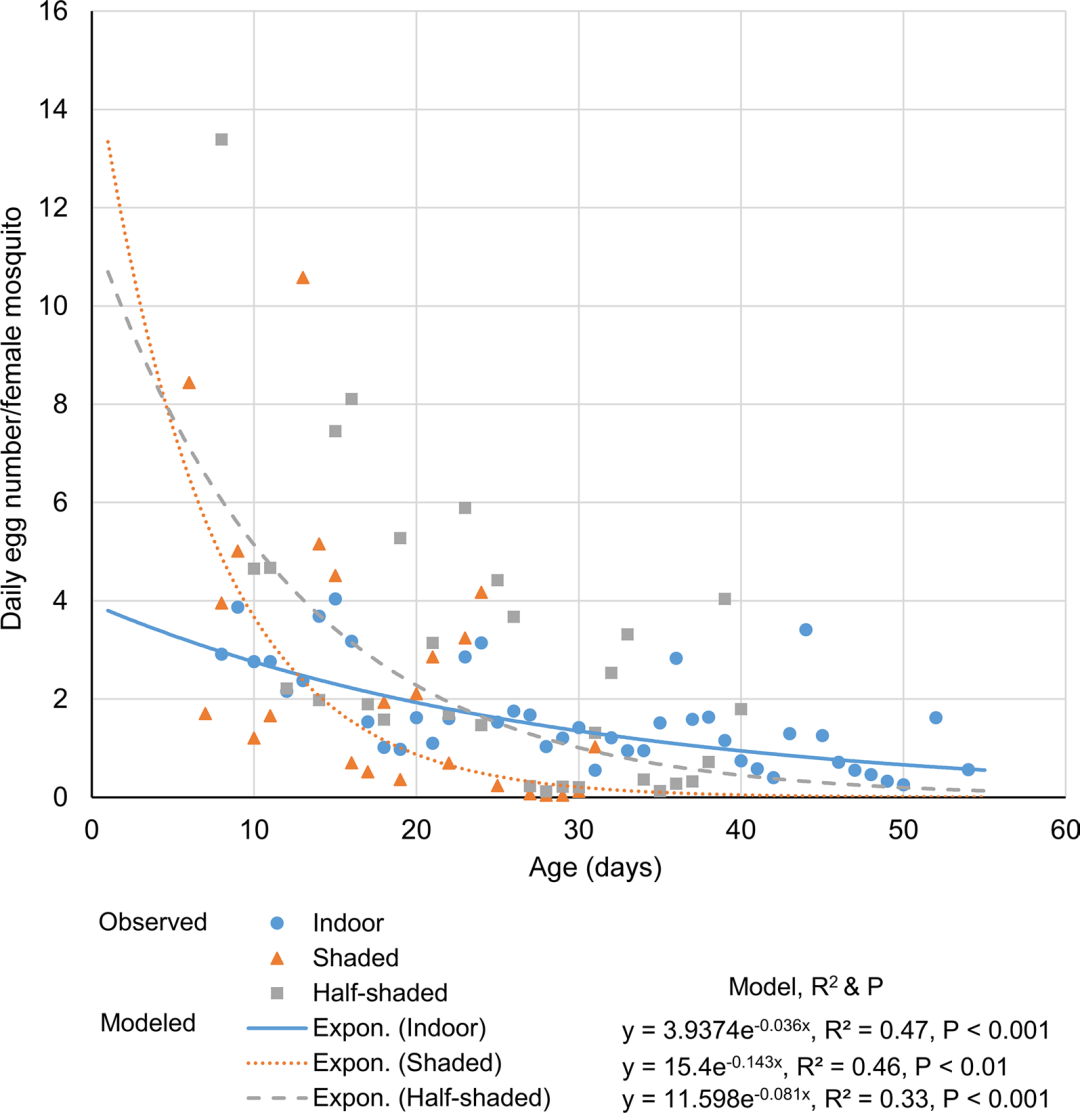
Age-specific *Aedes albopictus* egg mass in the three study settings. The value of *r*^2^ represents the proportion of variance in age-specific egg mass explained by age. *P* is the significance level of the goodness of fit

In addition, we found that female daily egg mass production was age-dependent in all settings (Fig. 6). Significant positive slope values (the rate of change in egg mass with age) in the model suggested that older mosquitoes had lower egg mass production than the younger ones (Fig. 6). The rates of change in egg mass with age were similar between the shaded and half-shaded settings (*t*-test, *t*_(50)_ = 1.57, *P* = 0.123, Fig. 6), but significantly lower under the indoor conditions compared to those in the half-shaded (*t*-test, *t*_(71)_ = 2.01, *P* < 0.05) and shaded (*t*-test, *t*_(65)_ = 3.22, *P* < 0.01) conditions (Fig. 6).

Overall, despite the differences in life span and reproductive duration of female mosquitoes in different environmental conditions, no significant difference was found in the mean net reproductive rates (*R*_0_) among these three settings (ANOVA, *F*_(2, 12)_ = 2.79, *P* = 0.101, Table 3). However, the per capita intrinsic growth rates (*r*) were significantly different among the three settings, with the highest in the shaded areas and lowest in the indoor setting (ANOVA, *F*_(2, 12)_ = 38.50, *P* < 0.0001; Tukey HSD, *P* < 0.05, Table 3).

## Discussion

Understanding the biology of mosquito vectors and exploiting their survival behavior in nature is important in implementing an integrated approach for the control and management of mosquitoes. Development times and survivorship of various stages of mosquitoes under different environments are of particular importance, as they affect the vectorial capacity, which is tightly linked to mosquito-borne disease transmission [42–45]. In our study, *Ae. albopictus* life trait parameters were established; age-specific mortality and age-specific egg mass models were constructed to predict the survivorship and fecundity capacity of the mosquito under different environmental conditions. Many of the details regarding *Ae. albopictus* female survivorship and reproduction under different environmental condition were reported for the first time. For example, female reproductive duration and daily mean reproduction have never been reported.

Indoor, outdoor, and different shading status in outdoor environments have different impacts on microclimate, such as temperature and humidity, which in turn affects vector ecology [10, 46–50]. In this study, we found that shading significantly increases humidity but reduces temperature. In contrast, indoor environments reduce humidity but have less impact on temperature. These differences may have profound impacts on larval development and adult survivorship and reproduction [30, 31, 33, 35]. Although the indoor average temperature of 29.7 °C was about 3 °C higher than that in the shaded area, temperatures under all three settings were within the range of optimal temperature for *Aedes* development and survival [37, 39, 46]. In addition, the average humidity was 75.6% in the indoor setting, which is within the WHO recommended range for laboratory mosquito rearing [51], whereas the humidity was close to 100% in all shaded settings, likely due the frequent rainfall in the study area, and may not be conducive for adult survival. Temperature and relative humidity are two of the most important abiotic factors influencing the development and survival of insects. Very high temperature may increase mosquito mortality [52]. Relative humidity can also influence mosquito abundance under field conditions [53, 54]. In this study, we observed constant high temperature in indoor settings, with an overall average of about 30 °C and maximum likely reaching 32 °C. However, adult females survived for the longest time, indicating that the impact of temperature alone on adult survival is limited. Further study is needed to illustrate how the combination of temperature and humidity impacts adult survivorship.

The most significant effect of shading is light intensity, with half-shading allowing for the strongest sunlight. In *Anopheles* studies, scientists have found that nearly full shading may completely prevent adult mosquito emergence, although the key driving factor is food availability [29, 33, 36]. In all laboratory experiments, there is usually a light/dark setting, for example, 12 h/12 h in a day or other similar settings [38, 39, 46]. In our indoor environment, we did not use any artificial light source, and thus relied solely on natural light. As a result, light intensity was very low, although the photoperiod with light was usually around 12–13 h. In the fully shaded area, light period and intensity were similar to indoors. In reality, there is daytime light in residential or office indoor environments, which may support larval development in container habitats such as flower pots. However, whether and to what extent these low light intensities affect larval development and pupation is not clear. Adding food to habitat water significantly increased pupation rate and accelerated larval development in all three conditions tested. In *Anopheles* experiments, shading was found to significantly reduce habitat biotic mass such as algae, which is the key food source for *Anopheles* larvae [36, 55]. The mechanisms by which low light intensity affects *Aedes* larval food sources warrant further investigation. Nonetheless, it is clear that shading status is critical for larval development.

Most of the previous life-table studies used artificial food as the larval food source [30, 31, 46]. A few studies found that food supply is a key factor in larval development and pupation. For example, Munga and colleagues found that in a forested area with nearly full shading, pupation was completely prevented without adding food to the artificial habitat. Furthermore, even in a half-shaded farmland area, the pupation rate can be significantly enhanced after adding food to the habitats [33], and adult development time is prolonged without adding food to habitats [29, 33]. Our results corroborate previous findings for *Aedes aegypti* [36, 48] and *Anopheles darlingi* [56].

Notably, we found little effect of water source (i.e. tap water vs. habitat water) on the larval life-table studies once additional food supplement was available. In most studies, especially in laboratory settings, tap water is standard for larval rearing. In our study, in addition to tap water, we used water collected from the habitats in the microcosms, which is similar to Munga and colleagues’ experiments [33]. In both cases, not adding food to the artificial habitats resulted in larval development times which were extremely long (no food vs. food: 28 days vs. 11 days) and pupation rates that were extremely low (0% vs. 20%) [33]. This finding of food limitation in natural habitat water is puzzling, as theoretically, habitat water should be able to support larval development. This result may be explained by the changes in biotic and abiotic content in the habitat water after the water was transferred from the field habitats to the artificial habitats where larvae were reared: the habitat water used in the experiments was no longer in the original habitat water. For example, in the case of *An. gambiae* experiments [33], the major food source, green algae, may not grow well in artificial habitats, and thus the food supply is disrupted [29, 36]. In addition, microbiological content may have changed after water was transferred to a semi-natural environment, which could reduce the food supply for *Ae. albopictus*. Altogether, these findings suggest that transferring natural habitat water into artificial habitat for larval rearing may fundamentally change the water biotic and abiotic content, which inhibits larval pupation. One potential solution may be to frequently replace the “used” habitat water with freshly collected natural habitat water. However, the effects of habitat water transfer on larval food supply and solutions for maintaining natural larval food supplies in habitat water need further investigation.

It is also worth noting the relatively long adult mosquito life span and reproductive period in our study. We found that female adult *Ae. albopictus* mosquitoes can live up to 70 days indoors and that reproduction can continue for more than 50 days. This information is extremely important in assessing the risk of *Aedes*-borne disease transmission. It has been reported that *An. gambiae* adults can live for up to 90 days indoors [31]. Thus, our finding of *Ae. albopictus* living for up to 70 days may be expected. In this study, the reproductive period of *Ae. albopictus* females in the shaded areas, whether half-shaded or fully shaded, was about 30 days and over 50 days, respectively, in the indoor environment; therefore, a very old female could still produce eggs, indicating the importance of old females. We believe this information is important when assessing disease transmission risk. However, the net reproductive rates of *Ae. albopictus* females were similar among the three settings, which indicated that both indoor and outdoor conditions in the study area can maintain *Ae. albopictus* population reproduction. This is the first field study to reveal that *Ae. albopictus* has similar reproductive capacity both indoors and outdoors.

We also reported age-dependent mortality rates and age-dependent reproductive capacity of adult *Ae. albopictus*. For both male and female *Ae. albopictus*, the mortality rates increased when they were aged, which is similar to that in *An. gambiae* and *Ae. aegypti* [57–59]. Likewise, female *Ae. albopictus* laid fewer eggs when they aged, a phenomenon consistent with a previous study [60]. We also found that the amount of lifetime egg mass of female *Ae. albopictus* in our study, conducted in a tropical area, was higher than that in a similar study conducted in Guangzhou, a subtropical city in southern China [34], potentially due to the difference in environmental and climatic conditions, since both were conducted in semi-natural conditions.

For life-table studies, regardless of whether they are conducted in laboratory controlled or semi-natural conditions, adult mosquitoes are usually fed glucose solution every day. Sugar solution serves as the basic food of adults [61]. Blood meals are also provided periodically to support the progeny, with protein for egg development. This may range from slightly to very different from natural mosquito diets, such as organic matter and protein from leaf/flower nectar or a blood meal source. The difference in the impact on adult survival is not clear between sugar-fed mosquitoes and mosquitoes fed with flower or leaf nectar. The current extent of our understanding is that adult mosquitoes’ primary food source is flower/leaf nectar, and female mosquitoes need protein (usually from a blood meal source) before they can lay eggs [62, 63]. In this study, the blood-feeding interval with mouse blood was 1 week, but it is unknown how the selected blood-feeding interval could affect adult survivorship and fecundity. Some studies report feeding of mosquitoes every day [59, 64], some every other day [57], and some once every 3 days [34]. The impact of the blood-feeding interval on mosquito survivorship and fecundity warrants further investigation. Furthermore, other studies on different mosquitoes have reported that the blood meal source affected the feeding and reproductive capacity of females [65–68]. The impact of different blood sources on *Ae. albopictus* survivorship and reproduction is unknown. However, since we used the same blood source and same feeding intervals during all experiments, the resulting female survivorship and reproductive characteristics are comparable within this study.

One limitation of this study is that we did not evaluate *Ae. albopictus* in fully open larval habitats. Most *Anopheles* studies have found that openness better supports larval development and larval pupation due to the direct sunlight, which increases the larval food supply [29, 33, 55]. However, summer in our study area is very hot, and temperatures under direct sunlight can easily reach 35 °C, which may not be conducive to larval development and adult reproduction of *Ae. albopictus* [35]. In addition, both *Ae. albopictus* larvae and adult mosquitoes have been found to hide to avoid direct expose to sunlight [13, 69, 70]. Therefore, conducting life-table experiments under direct sunlight may not be necessary, as *Ae. albopictus* would likely avoid these habitats. In fact, almost no *Anopheles* studies have been conducted in fully open areas [30, 31].

Another limitation is the study sites used. In our study, only one indoor, shaded, and half-shaded site each were used. This may compromise the generalizability of the findings. However, as we conducted five replications of experiments at each site, the conclusion of the study may not be significantly affected. Nonetheless, a more systematic study should be conducted in the future to explore the impact of habitat conditions on mosquito ecology.

## Conclusion

The results of this study indicate that different environmental conditions affect *Ae. albopictus* immature development time and adult emergence rates, as well as adult mosquito survivorship and reproduction. Life-table experiments in different ecological settings is one way to examine these relationships. Although its design is perhaps oversimplified, it reduces other confounding factors. Overall, a half-shaded environment provides potentially the most conducive conditions for *Ae. albopictus* larval development, adult emergence rates, and female reproduction, although adult mosquitoes may live longer indoors. Our findings provide insight into the potential influence of indoor and shading conditions on the biology of *Ae. albopictus*.

## Supplementary Information

**Additional file 1: Figure S1**. Environments tested in our study areas. **a**, **d** and **g** Indoor setting. **b**, **e** and **h** Half-shaded setting. **c**, **f** and **i** Shaded setting.

## Data Availability

All data generated or analyzed during this study are included in this published article and its supplementary information files.
