# Supplementary figures and images for: Aedes albopictus life table: environment, food, and age dependence survivorship and reproduction in a tropical area
Source: Parasit Vectors. 2021 Nov 7;14:568. doi: 10.1186/s13071-021-05081-x (PMC8573987; doi:10.1186/s13071-021-05081-x)

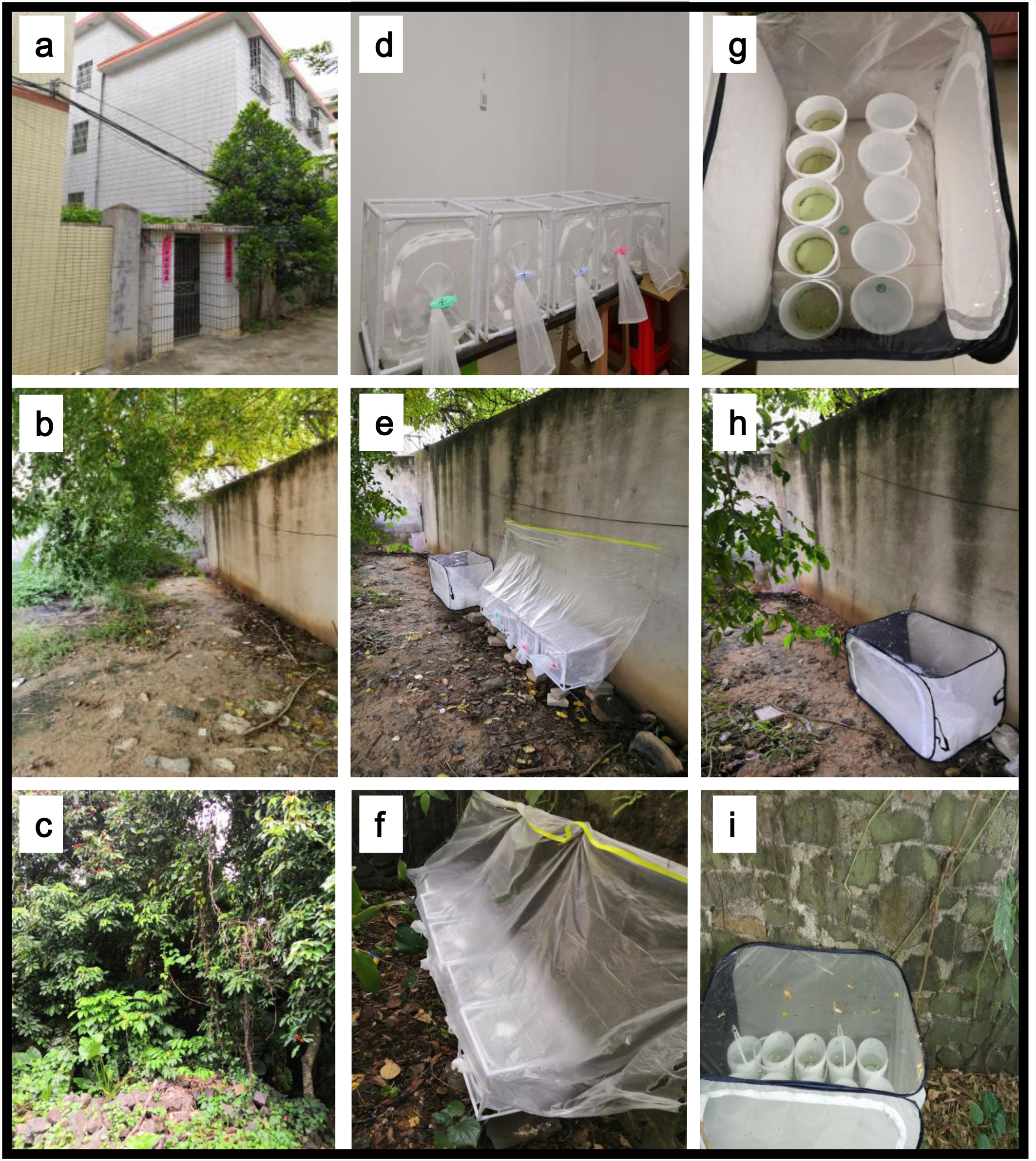

Supplement: Supplementary file 1 — Additional file 1: Figure S1. Environments tested in our study areas. a, d and g Indoor setting. b, e and h Half-shaded setting. c, f and i Shaded setting. [file 13071_2021_5081_MOESM1_ESM.tif]
